# Nicotine-induced bullous fixed drug eruption

**DOI:** 10.1016/j.jdcr.2022.08.041

**Published:** 2022-08-27

**Authors:** Brynn Sargent, Lina Saeed, Dani Zhao, Anna-Marie Hosking, Hadas Skupsky, Maryam Safaee

**Affiliations:** a School of Medicine, University of California, Irvine; b Department of Dermatology, University of California, Irvine

**Keywords:** Bullous disease, fixed drug eruption, nicotine, EM, Erythema multiforme, FDE, Fixed drug eruption, BP, Bullous pemphigoid

## Introduction

Fixed drug eruption (FDE) is a relatively common cutaneous drug reaction, occurring in up to 22% of patients experiencing drug-induced skin necrosis.^1^ Historically, the most common inciting medications include various antibiotic agents and non-steroidal anti-inflammatory drugs.^1^ Previous reports have also identified non-medication causes, such as certain food items. FDE skin lesions are classically described as violaceous circular patches, plaques, or bullae that recur at the same site with each successive administration of the causative agent.^1^ Distribution of lesions can vary across subtypes of FDE. However, it most commonly affects areas with thin skin, such as the lip mucosa, genitals, and perianal sites.^1^ One study found that as many as 90% of men with FDE present with lesions on the genitals.^2^ In this report, we describe a case of nicotine-induced bullous FDE.

## Case report

A 35-year-old man with a 5-year history of recurrent mucocutaneous blisters presented to our clinic with intermittent flares over the preceding years. The patient presented with blisters progressing to ulcers on the tongue, scrotum, elbow, and dorsal fingers. No clear inciting trigger had been identified previously. Previous workup, including multiple punch biopsies, had revealed subepidermal vesiculation, interface dermatitis, and mixed inflammatory infiltrates with negative direct immunofluorescence (DIF) studies. Previous serology studies showed mildly elevated bullous pemphigoid (BP180 and BP230) antibody titers. Differential diagnosis included erythema multiforme (EM), FDE, and atypical BP. The patient failed to improve on dapsone and valacyclovir for possible EM and two infusions of rituximab for possible BP. While his disease was adequately controlled on high-dose oral prednisone, this course of treatment was unsustainable given the risks associated with chronic high-dose steroid use. His medication regimen at presentation included mycophenolate mofetil 1500 mg daily, doxycycline 100 mg twice daily, and niacinamide 500 mg twice daily. However, the patient had been unable to take these medications because of significant pain from his tongue ulcers. His social history was significant for intermittent cigarette use, and the patient had smoked a pack of cigarettes one week prior to this presentation.

**Fig 1:**
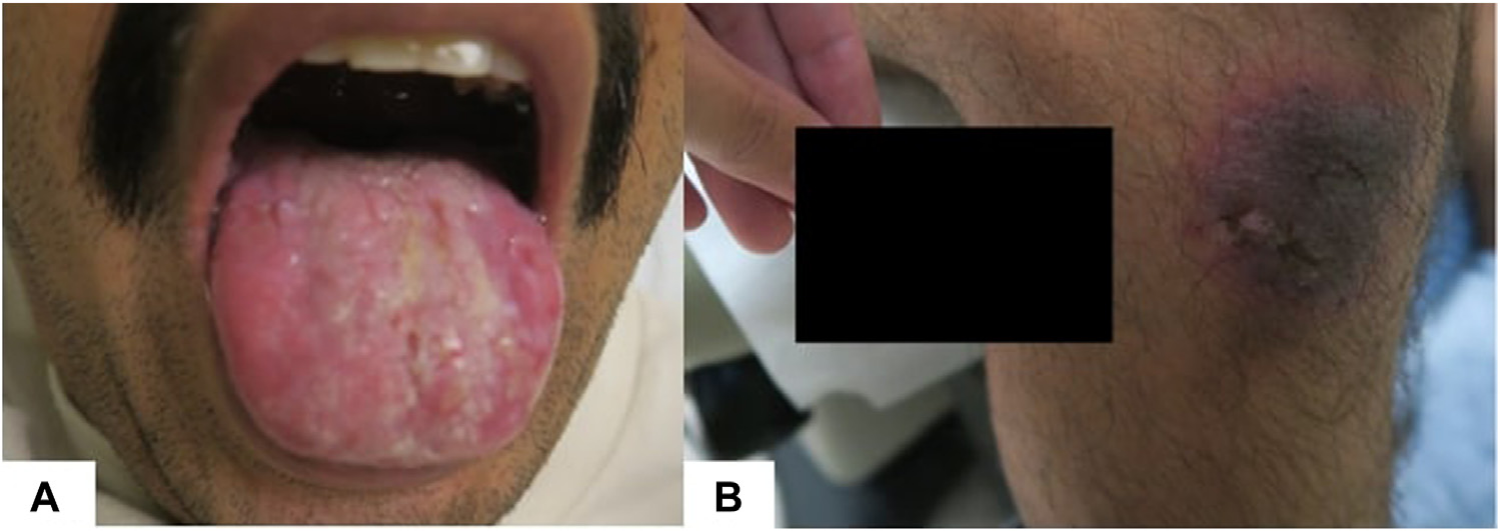
Ulcers on the tongue (**A**) and dusky eroded plaque on the knee (**B**).

**Fig 2:**
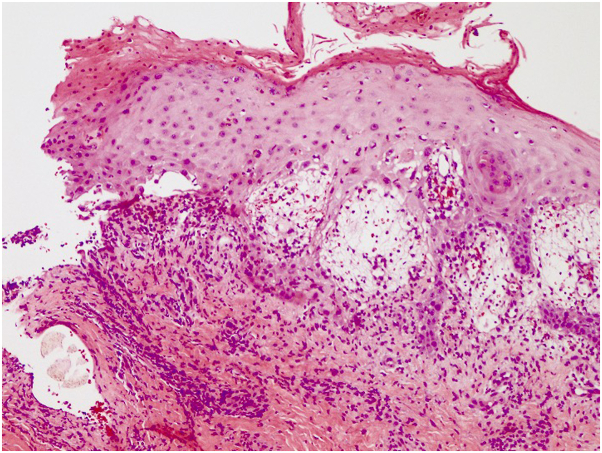
Interface dermatitis with prominent dyskeratosis and subepidermal vesiculation (hematoxylin-eosin staining, original magnification ×100).

The patient was afebrile with stable vital signs. Physical examination revealed numerous ulcerations on the tongue, scrotum, glans penis, elbow, and bilateral dorsal fingers without ocular or nasal mucosal lesions (Fig 1). Laboratory tests revealed no leukocytosis. Given his inability to eat and drink because of severe intraoral pain, the patient was admitted for pain management and wound care and was started on topical clobetasol and viscous oral lidocaine, valacyclovir 500 mg twice daily, and intravenous methylprednisolone 48 mg daily. Blood and urine culture, respiratory viral panel, and HIV testing were all negative. A shave biopsy of the dorsal right 3^rd^ finger showed interface dermatitis with prominent dyskeratosis and subepidermal vesiculation (Fig 2). A direct immunofluorescence study was negative. While this biopsy had features similar to those taken during previous flares, it also demonstrated a lichenoid interface dermatitis with prominent necrosis and full-thickness epidermal dyskeratosis. The inflammatory infiltrate included lymphocytes, histiocytes, and neutrophils with no significant involvement of eosinophils, though eosinophils were present on some of the previous biopsies. The presence of neutrophils within the inflammatory infiltrate favored a diagnosis of bullous FDE in the clinical context of cigarette exposure, which had been a consistent symptomatic trigger throughout this patient’s history. Over the next two days, the patient showed significant improvement without development of new lesions, and he was discharged on a six-week prednisone taper. During discussion with the patient, it was noted that he had abstained from smoking prior to the flare and then had developed significant mucocutaneous blistering upon reinitiating tobacco use. Further history revealed that the unifying trigger to all his flares was the exposure to nicotine, whether in the form of tobacco, chewing gum, or patches. We herein describe a unique case of nicotine-induced bullous FDE.

## Discussion

Histopathologic examination of skin biopsy in FDE typically reveals subepidermal vesiculation with hydropic degeneration of the basal layer and scattered necrotic keratinocytes within the epidermis.^3^ Interface dermatitis is frequently present with an inflammatory infiltrate typically consisting of lymphocytes and eosinophils.^4^^,^^5^ Macrophage phagocytosis of melanosomes from necrosed keratinocytes causes pigmentary incontinence, resulting in hyperpigmented macules clinically following resolution of acute skin lesions.^3^

The histologic differential diagnosis of FDE commonly includes other interface dermatitides with prominent dyskeratosis, including EM and Stevens-Johnson syndrome/toxic epidermal necrolysis. There is significant histologic overlap of these conditions, although FDE typically presents with a more mixed inflammatory infiltrate, including eosinophils and sometimes neutrophils.^3^ Clinical features can also help to differentiate among these related entities. Necrotic lesions in FDE can closely resemble the classic targetoid lesions of EM. However, close examination of FDE lesions will not reveal the typical targetoid lesions, composed of three distinct zones, observed in EM. While severe forms of generalized bullous FDE can closely resemble Stevens-Johnson syndrome/toxic epidermal necrolysis, the former often lacks the constitutional symptoms and severe systemic effects of Stevens-Johnson syndrome/toxic epidermal necrolysis.^6^

Despite having no exposure to the classic causative medications, we believe the combined clinicopathologic findings in this case represent a nicotine-induced FDE. Throughout his five-year history of symptoms, the patient has consistently experienced relapse within days to a week of resuming smoking or using other nicotine-containing products, such as gum or patches, even in the absence of smoking. His lesions frequently occur on similar areas of the body, though his episodes have demonstrated minor variability in lesion distribution along the extremities. His lesions to date have not developed the characteristic hyperpigmentation following resolution of acute exacerbation; however, cases of non-pigmenting FDE have previously been reported.^7^ The patient has failed to develop significant systemic effects during these episodes, a finding that also favors FDE over the other entities discussed above.

Multiple biopsies taken over the last several years have demonstrated subepidermal vesiculation and the presence of an interface dermatitis with dyskeratosis. The inflammatory infiltrate did differ slightly in composition from the classical presentation of FDE, in that the number of neutrophils, and not eosinophils, were increased. However, previous reports have noted an increase in neutrophils within FDE lesions.^8^^,^^9^ Lastly, although the patient experienced a reduction in flares while taking maintenance high-dose prednisone, his only exacerbation-free periods were observed during total nicotine abstinence. While topical or systemic steroid treatment is often administered during acute exacerbations of FDE, causative drug avoidance is the mainstay of treatment.^10^ We hope this case report serves to further expand the consideration of possible causative agents for FDE.

## Conflicts of interest

None disclosed.
